# Glycoprotein IIb/IIIa inhibitors use in the setting of primary percutaneous coronary intervention for ST elevation myocardial infarction in patients pre‐treated with newer P2Y12 inhibitors

**DOI:** 10.1002/clc.23654

**Published:** 2021-06-11

**Authors:** Katrien Blanchart, Thibaut Heudel, Pierre Ardouin, Adrien Lemaitre, Clément Briet, Mathieu Bignon, Rémi Sabatier, Damien Legallois, Vincent Roule, Farzin Beygui

**Affiliations:** ^1^ CHU de Caen Normandie, Service de Cardiologie Caen France; ^2^ Normandie Univ, UNICAEN, EA 4650 Signalisation, électrophysiologie et imagerie des lésions d'ischémie‐reperfusion myocardique Caen France

**Keywords:** acute coronary syndrome, glycoprotein IIb/IIIa inhibitors, P2Y12 inhibitors, prasugrel, ST segment elevation myocardial infarction, ticagrelor

## Abstract

**Objectives:**

We sought to investigate the safety and potential benefit of administrating glycoprotein IIb‐IIIa inhibitors (GPIs) on top of more potent P2Y12 inhibitors.

**Background:**

A number of clinical trials, performed at a time when pretreatment and potent platelet inhibition was not part of routine clinical practice, have documented clinical benefits of GPI in ST‐segment elevation myocardial infarction (STEMI) patients at the cost of a higher risk of bleeding.

**Methods:**

We used the data of a prospective, ongoing registry of patients admitted for STEMI in our center. For the purpose of this study only patients presenting for primary percutaneous coronary intervention and pretreated with new P2Y12 inhibitors (prasugrel or ticagrelor) were included. We compared patients who received GPI with those who did not.

**Results:**

Eight hundred twenty‐four STEMI patients were included in our registry; GPIs were used in 338 patients (41%). GPI patients presented more often with cardiogenic shock and Thrombolysis in myocardial infarction (TIMI) flow grade <3. GPI use was not associated with an increase in in‐hospital or 3‐month mortality. Bleeding endpoints were similar in both groups.

**Conclusions:**

Our study suggests that GPI may be used safely in combination with recent P2Y12 inhibitors in STEMI patients in association with modern primary percutaneous coronary intervention strategies (radial access and anticoagulation with enoxaparin) with similar bleeding and mortality rates at hospital discharge and 3‐month follow‐up.

## 1 INTRODUCTION

Fast and potent platelet inhibition is recommended at the time of percutaneous coronary intervention (PCI) in patients with ST segment elevation myocardial infarction (STEMI) in order to restore coronary flow as soon as possible.^1^

Glycoprotein IIb/IIIa inhibitors (GPIs), currently recommended only in highly thrombotic situations,^1^ have longtime been used on a routine basis in the setting of primary PCI, when pretreatment with oral potent platelet inhibition was not part of routine clinical practice, with documented clinical benefits patients but also an increased risk of bleeding.^2^, ^3^, ^4^ In the era of more potent and faster acting P2Y12‐inhibitors like prasugrel and ticagrelor, the benefit and, more importantly, the safety of GPI, remains discussed. Moreover, the recommended use of GPIs in the presence of no‐reflow or thrombotic complications (Class IIa, level of evidence C) and in P2Y12‐inhibitor naïve patients undergoing PCI (Class IIb, level of evidence C)^1^, ^5^ is not supported by any randomized controlled trial. Data concerning the use of GPI in association with prasugrel and ticagrelor are scarce and limited to sub‐analyses of original publications with mostly femoral approaches.

We sought to investigate the safety and efficacy of GPI administration in association with ticagrelor or prasugrel as recommended by guidelines,^1^ in the setting of primary PCI in a prospective real‐life cohort of STEMI patients in an experienced radial‐first center.

## 2 METHODS

### 2.1 Study population and protocol

The study is based on data from a prospective, observational, open, ongoing registry of patients included in a regional multicenter STEMI network and admitted in our center for primary PCI.

Follow‐up is performed by a dedicated staff at hospital discharge and, at 3 months, by a physical visit or phone call to the patients, relatives or patients' physician. In case of hospitalization, medical records are requested and reviewed. When data are unavailable, the national mortality database is consulted to assess vital status. All clinical data are prospectively recorded.

The study was approved by the French North‐West‐3 ethics board. All patients gave informed consent to be included and followed up.

For the purpose of our study we only included patients who received pretreatment with newer, potent P2Y12 inhibitors (prasugrel or ticagrelor). Patients receiving fibrinolytic treatment were excluded.

### 2.2 Pharmalogical treatment

All patients were pretreated following the regional STEMI protocol with enoxaparin 0.5 mg/kg intravenous (iv), aspirin (250 mg intravenous bolus) and P2Y12 inhibitor oral loading doses (ticagrelor 180 mg, prasugrel 60 mg) prior to coronary angiography.

GPI treatment could be considered at the discretion of the operator for bail‐out (no reflow or thrombotic complications) or in case of high thrombus burden after angiography was performed. Abciximab (0.25 mg/kg iv‐bolus, 0.125 μg/kg/min infusion for 12–36 h), tirofiban (25 μg/kg over 3 min followed by 0.15 μg/kg/min infusion for 18–24 h) or eptifibatide (180 μg/kg iv‐bolus repeated after 10 min followed by 2 μg/kg/min infusion for 18–96 h) could be used. Doses were adapted to renal function if needed.

### 2.3 Study Outcomes and definitions

Routine (or planned) GPI use was defined as initiation of GPI infusion prior to PCI guidewire insertion in patients with heavy thrombotic burden, in opposition to bail‐out use, where GPI was used after initiation of PCI for the management of angiographic or clinical complications. Cardiogenic shock was defined as persistent systolic hypotension <100 mmHg requiring the use of inotropic drugs.

Bleeding events were classified using the Bleeding Academic Research Consortium (BARC) definition.^6^ The primary efficacy endpoint was all‐cause in hospital‐mortality. The primary safety outcome of our study was the onset of in‐hospital major bleeding defined as BARC ≥2.

Secondary endpoints included 3‐month all‐cause mortality and major bleeding, radiologically confirmed stroke, recurrent MI,^7^ probable or definite stent thrombosis, rehospitalization for cardiovascular causes and final post‐PCI TIMI flow grade and minor bleeding, defined as BARC <2.

### 2.4 Statistical analysis

We divided the patients into two groups: Those who received GPI (routine or bail‐out) and those who did not.

Continuous and categorical variables were expressed as mean ± SD and numbers (%), respectively. The distribution of variables was visually assessed. Continuous variables were all normally distributed and compared between groups by the Student's *t* test. Categorical variables were compared using the *χ* ^2^ or Fisher's exact tests when adequate.

We calculated a propensity score to account for the pre‐PCI propension of patients to receive GPI using a logistic regression model including pre‐defined variables: age, gender, prior PCI, known renal failure, oral anticoagulant treatment, pretreatment with aspirin, prasugrel or ticagrelor, stent thrombosis at presentation, mechanical thrombus aspiration, and initial TIMI flow grade. We also performed a post hoc complementary analysis using a secondary propensity score including variables diabetes, body mass index and cardiogenic shock, on top of those included in the latter model.

The association between GPI use and 3‐month mortality was estimated using unadjusted and adjusted Cox regression models with calculation of HRs (95% CI). All other outcomes were compared between groups using unadjusted and adjusted logistic regression models with calculation of ORs (95% CI). All models were adjusted on the propensity score, age and gender. GRACE^8^ and CRUSADE^9^ scores were forced into the models for the assessment of mortality and bleeding, respectively.

We also performed a propensity score tertile‐matched analysis using similar models.

All tests were 2‐sided and *p* < .05 was considered to be significant.

R software version 3.0.0 (2013‐04‐03) for MacOS (R Foundation for Statistical Computing) was used for statistical analyzes.

## 3 RESULTS

**FIGURE 1:**
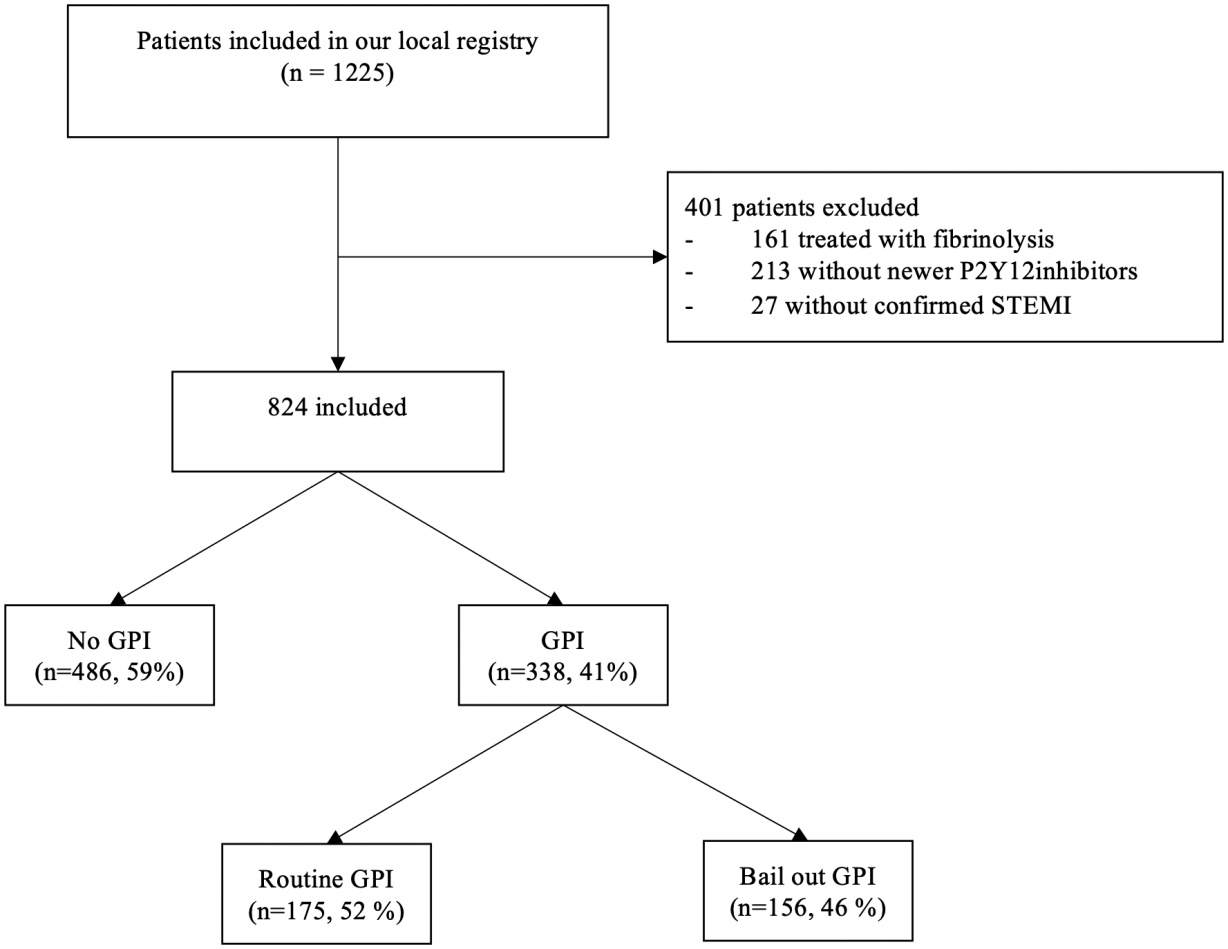
Flowchart. STEMI, ST segment elevation myocardial infarction; GPI, glycoprotein IIb‐IIIa inhibitor

Among 1225 patients prospectively included in our database between July 2015 and December 2018, 401 were excluded (161 with fibrinolysis, 213 without newer P2Y12‐inhibitor pre‐treatment, 27 without confirmed STEMI diagnosis). A total of 824 STEMI patients were finally included for the current analysis. GPI was used in 338 patients (41%) (Figure 1). Tirofiban, eptifibatide, and abciximab were used in 95.3%, 3.3%, and 1.2% of patients, respectively.

The indication for GPI use was routine in 175 (52%) and “bail‐out” in 156 (46.2%) patients.

### 3.1 Patient characteristics (Table 1)

Table 1 summarizes baseline characteristics of the studied population. Mean age was 62.1 ± 13.1 years and 76% of patients were men.

**TABLE 1:** Baseline characteristics for the overall study population and the matched cohort according to the use or not of GPI

	Overall study population				Propensity‐matched Cohort		
	All 824 (100%)	No GPI *N* = 486 (59%)	GPI *N* = 338 (41%)	*p* value	No GPI *N* = 338	GPI *N* = 338	*p* value
Demographics							
Age	62.09 ± 13.13	63.39 ± 13.38	60.22 ± 12.55	<.01	62.35 ± 12.96	60.22 ± 12.55	.03
Female	199 (24%)	121 (24.9%)	78 (23.1%)	.55	80 (23.7%)	78 (23.1%)	.87
Height	171.02 ± 9.37	170.42 ± 9.51	171.88 ± 9.1	.03	169.86 ± 9.43	171.39 ± 9.35	.04
Weight	78.69 ± 15.51	77 .21 ± 14.44	80.80 ± 16.72	<.01	77.50 ± 14.07	80.95 ± 16.94	<.01
BMI	26.82 ± 4.82	26.52 ± 4.55	27.25 ± 5.17	.04	26.71 ± 4.77	27.25 ± 5.17	.17
Past medical history							
Hypertension	342 (42%)	214 (44%)	128 (37.9%)	.08	144 (42.6%)	128 (37.9%)	.33
Dyslipidemia	312 (38%)	171 (35.2%)	141 (41.7%)	.06	122 (36.1%)	141 (41.7%)	.24
Current smoker	460 (56%)	255 (52.5%)	205 (60.7%)	.02	190 (56.2%)	205 (60.7%)	.45
Diabetes mellitus	110 (13%)	57 (11.7%)	53 (15.7%)	.10	42 (12.4%)	53 (15.7%)	.26
Family history of heart or cardiovascular disease	190 (23%)	119 (24.5%)	71 (21%)	.24	84 (24.9)	71 (21.0)	.30
Prior CABG	2 (0%)	1 (0.2%)	1 (0.3%)	.80	1 (0.3)	1 (0.3)	1.00
Prior PCI	88 (11%)	42 (8.6%)	46 (13.6%)	.02	32 (9.5)	46 (13.6)	.11
Prior stroke	25 (3%)	15 (3.1%)	10 (3%)	.92	10 (3.0)	10 (3)	1.00
Kidney failure	17 (2%)	13 (2.7%)	4 (1.2%)	.14	8 (2.4)	4 (1.2)	.26
VKA treatment	6 (1%)	6 (1.2%)	0 (0%)	.04			
DOAC treatment	2 (0%)	2 (0.4%)	0 (0%)	.24			
Presentation							
SBP	145.8 ± 47.5	146.8 ± 44.0	144.4 ± 52.2	.50	154.1 ± 41.6	156.4 ± 47.8	.50
DBP	72.4 ± 29.3	75.9 ± 27.8	67.3 ± 30.7	<.01	75.5 ± 27.2	67.3 ± 30.6	<.001
Pulse	68.6 ± 24.1	70.6 ± 22.7	65.9 ± 25.7	<.01	74.0 ± 27.7	70.0 ± 28.9	.06
Killip ≥2	166 (20.1%)	92 (18.9%)	74 (21.9%)	.17	65 (19.7%)	74 (21.9%)	.38
Cardiogenic shock	54 (7%)	21 (4.3%)	33 (9.8%)	<.01	14 (4.1%)	33 (9.8%)	<.01
CPA	89 (11%)	49 (10.1%)	40 (11.8%)	.43	36 (10.7%)	40 (11.8%)	.65
CPA at presentation	44 (5%)	22 (4.5%)	22 (6.5%)	.21	17 (5.0%)	22 (6.5%)	.42
CPA during PCI	11 (1%)	3 (0.6%)	8 (2.4%)	.03	3 (0.9%)	8 (2.4%)	.15
Time from chest pain onset to PCI	341.8 ± 300.7	326.8 ± 280.4	363.3 ± 326.9	.10	321.2 ± 277.4	363.3 ± 326.9	.11
Time from ECG to PCI (min)	140.6 ± 184.2	142.0 ± 187.2	138.6 ± 180.0	.79	142.5 ± 191.9	138.6 ± 180.0	.78
Peri procedural Treatment							
Aspirin pretreatment	808 (98%)	481 (99%)	327 (96.7%)	.02	333 (98.5%)	327 (96.7%)	.82
Ticagrelor pretreatment	621 (75%)	384 (79%)	237 (70.1%)	<.01	256 (75.7%)	237 (70.1%)	.39
Prasugrel pretreatment	203 (25%)	102 (21%)	101 (29.9%)	<.01	82 (24.3%)	101 (29.9%)	.16
LMWH	814 (99%)	480 (98.8%)	334 (98.8%)	.95	334 (98.8%)	334 (98.8%)	1.00
UFH	43 (5%)	26 (5.3%)	17 (5%)	.84	16 (4.7%)	17 (5.0%)	.86
GPI treatment	338 (41%)	0 (0%)	338 (100%)				
Abciximab	4 (0%)	0 (0%)	4 (1.2%)				
Eptifibatide	11 (1%)	0 (0%)	11 (3.3%)				
Tirofiban	322 (39%)	0 (0%)	322 (95.3%)				
Planned GPI use	175 (21%)	0 (0%)	175 (51.8%)				
Bail out GPI use	156 (19%)	0 (0%)	156 (46.2%)				
Angiographic characteristics							
Culprit coronary artery							
Right coronary artery	331 (40%)	189 (38.9%)	142 (42%)	.37	140 (41.4%)	142 (42.0%)	.91
Left anterior descending artery	351 (43%)	213 (43.8%)	138 (40.8%)	.39	142 (42.0%)	138 (40.8%)	.81
Left circumflex artery	122 (15%)	75 (15.4%)	47 (13.9%)	.54	53 (15.7%)	47 (13.9%)	.55
Stent thrombosis	8 (1%)	1 (0.2%)	7 (2.1%)	<.01	1 (0.3%)	7 (2.1%)	.07
Thrombectomy	212 (26%)	60 (12.3%)	152 (45%)	<.01	60 (17.8%)	152 (45.0%)	<.01
Access site							
Transradial	806 (98%)	483 (98%)	323 (97.6%)	.71	324 (97.9)	323 (97.6)	.97
Transfemoral	17 (2%)	9 (1.8%)	8 (2.4%)	.56	6 (1.8)	8 (2.4)	.59
Basal TIMI flow Grade 3	199 (24.2%)	160 (32.9%)	39 (11.5%)	<.01	84 (24.9%)	39 (11.5%)	<.01
Final TIMI flow grade 3	746 (90.5%)	441 (90.7%)	305 (90.2%)	.81	305 (90.2%)	305 (90.2%)	1
Stent characteristics							
Number of stents	1.3 ± 0.7	1.2 ± 0.7	1.3 ± 0.8	.03	1.3 ± 0.7	1.3 ± 0.8	.15
Total length (mm)	30.7 ± 15.1	29.4 ± 13.6	32.7 ± 16.8	<.01	29.5 ± 14.3	32.2 ± 17.2	.03
Diameter (mm)	3.0 ± 0.5	3.0 ± 0.5	3.1 ± 0.5	<.01	3.0 ± 0.48	3.1 ± 0.5	<.001
Admission biological and echocardiographic characteristics							
Creatinine (μmol/L)	90.8 ± 80.0	91.2 ± 91.5	90.2 ± 59.9	.85	91.4 ± 82.1	90.5 ± 67.4	.88
Hematocrit	41.9 ± 5.2	42.1 ± 5.2	41.7 ± 5.2	.34	41.3 ± 5.1	41.3 ± 5.2	.91
LVEF (%)	47.6 ± 11.9	47.9 ± 11.9	47.3 ± 12.0	.44	50.6 ± 9.3	49.0 ± 10.7	.05
Risk score							
GRACE score	155.79 ± 43.19	155.39 ± 41.24	156.38 ± 45.90	.75	153.8 ± 40.0	156.4 ± 45.9	.43
CRUSADE score	23.85 ± 11.13	23.17 ± 11.23	24.83 ± 10.93	.03	22.86 ± 11.25	24.8 ± 10.9	.02

Abbreviations: CABG, coronary artery bypass grafting; CPA, cardiopulmonary arrest; DBP, diastolic blood pressure; DOAC, direct oral anticoagulant; GPI, glycoprotein IIb‐IIIa inhibitor; LVEF, left ventricular ejection fraction; LMWH, low‐molecular‐weight heparin; PCI, percutaneous coronary intervention; SBP, systolic blood pressure; UFH, unfractionated heparin; VKA, vitamin K antagonist.

Patients treated with GPI were younger (*p* < .001), had higher BMI (*p* = .04), more frequently a current smoker status (60.7% vs. 52.5%, *p* = .02) and prior PCI (13.6% vs. 8.6%, *p* = .02). Unlike those without GPI, no patient in the GPI group had oral anticoagulant treatment.

Systolic blood pressure (SBP) was lower (*p* < .01) and rates of cardiogenic shock were twice as high in the GPI group (9.8% vs. 4.3% *p* = .002). Cardiac arrest during PCI was also more frequent in the GPI group (2.4% vs. 0.6%, *p* = .03).

First medical contact (defined by the qualifying ECG) to balloon times were similar between the two groups. Pretreatment with ticagrelor was less frequent (70.1% vs. 79.0%, *p* = .004), whereas pretreatment with prasugrel was more frequent in the GPI group (29.9% vs. 21.0%, *p* = .003).

Arterial access site was predominantly radial (overall 98%) and equally distributed between the two groups.

GPI patients were more likely to be admitted for a stent thrombosis (2.1% vs. 0.2%, *p* = .007) and to be treated with thrombus aspiration (45.0% vs. 12.3%, *p* < .001).

The number, length and average diameter of stents were significantly higher in the GPI group.

Baseline rates of TIMI flow Grade 3 were lower in the GPI group (11.5% vs. 32.9%, *p* < .001) but final post‐PCI TIMI flow Grade 3 rates were similar between groups (90.2% vs. 90.7%, *p* = .81).

Other characteristics were comparable between groups.

Six hundred and seventy‐six patients were included in the propensity‐matched cohort to account for the pre‐PCI propension of patients to receive GPI.

### 3.2 Outcomes (Tables 2 and 3)

All patients completed 3‐month follow‐up. Thirty‐five (4.2%) deaths were recorded at hospital discharge and 46 (5.6%) at 3‐month follow‐up. All outcomes were similarly distributed between groups at hospital discharge and 3‐month follow‐up in both global and propensity‐matched analyses.

In‐hospital and 3‐month mortality was similar in the two groups and was mostly cardiovascular related in both groups (Table 2).

**TABLE 2:** Primary and secondary endpoints in the overall study population and the propensity‐matched population

Outcomes	Overall study population				Propensity‐matched Cohort		
	All 824 (100%)	No GPI *N* = 486 (59%)	GPI *N* = 338 (41%)	*p* value	No GPI *N* = 338	GPI *N* = 338	*p* value
In‐hospital mortality	35 (4.2%)	17 (3.5)	18 (5.3)	.20	11 (3.3)	18 (5.3)	.20
In‐hospital CV mortality	35 (4.2%)	17 (3.5)	18 (5.3)	.20	11 (3.3)	18 (5.3)	.20
In‐hospital major bleeding	11 (1.3%)	8 (1.6)	3 (0.9)	.35	7 (2.1)	3 (0.9)	.22
3‐month mortality	46 (5.6%)	26 (5.3)	20 (5.9)	.73	17 (5.0)	20 (5.9)	.62
3‐month CV mortality	37 (4.5%)	18 (3.7)	19 (5.6)	.19	11 (3.3)	19 (5.6)	.14
3‐month major bleeding	23 (2.8%)	16 (3.3)	7 (2.1)	.30	12 (3.6)	7 (2.1)	.26
In‐hospital mortality or bleeding	43 (5.2%)	24 (4.9)	19 (5.6)	.66	17 (5.0)	19 (5.6)	.74
3‐month mortality or bleeding	64 (7.8%)	40 (8.2)	24 (7.1)	.55	27 (8.0)	24 (7.1)	.67
Minor bleeding	5 (0.6%)	3 (0.6)	2 (0.6)	.96	1 (0.3)	2 (0.6)	.57
Stroke	11 (1%)	6 (1.2)	5 (1.5)	.76	6 (1.8)	5 (1.5)	.76
New ACS	27 (3%)	15 (3.1)	12 (3.6)	.71	13 (3.8)	12 (3.6)	.84
Definite or probable stent thrombosis	11 (1%)	4 (0.8)	7 (2.1)	.12	2 (0.6)	7 (2.1)	.12
Definite stent thrombosis	7 (1%)	2 (0.4)	5 (1.5)	.10	1 (0.3)	5 (1.5)	.14
Hospitalization CV	30 (4%)	14 (2.9)	16 (4.7)	.16	10 (3.0)	16 (4.7)	.24

*Note*: There was only one patient presenting intracranial bleeding (GPI group, intracranial bleeding was in‐hospital).

Abbreviations: ACS, acute coronary syndrome; CV, cardiovascular.

Major in‐hospital and 3‐month bleeding was not different between groups (0.9% vs. 2.1%, *p* = .22, 2.1% vs. 3.6%, *p* = .26) with only one patient presenting in‐hospital intracranial bleeding (in the GPI group). Minor bleeding was similar between GPI and no‐GPI patients (0.6% vs. 0.3%, *p* = .57). In hospital and 3 month major bleeding were not different in the logistic regression model between GPI and no‐GPI patients (OR: 0.43 [0.11; 1.66], *p* = .22, HR 0.58 [0.23; 1.48], *p* = .26).

The sensitivity post hoc analysis using the secondary propensity model provided similar matching and consistent results as reported in Table 3.

**TABLE 3:** Regression models with GPI as a 3‐category variable

Outcome	Overall population				Matched cohort		Post hoc complementary matched analysis	
	Univariable		Multivariable		Univariable		Univariable	
	HR/OR	*p*	HR/OR	*p*	HR/OR	*p*	HR/OR	*p*
In‐hospital mortality	1.55 [0.78; 3.08]	.20	1.39 [0.52; 3.72]	.51	1.64 [0.77; 3.46]	.20	1.64 [0.77; 3.47]	.20
In‐hospital cardiovascular mortality	1.55 [0.78; 3.08]	.20	1.39 [0.52; 3.72]	.51	1.64 [0.77; 3.46]	.20	1.64 [0.77; 3.46]	.20
In‐hospital major bleeding	0.54 [0.12; 1.86]	.36	0.61 [0.12; 2.43]	.51	0.43 [0.11; 1.66]	.22	0.50.[0.13; 2.00]	.33
3‐month mortality	1.13 [0.63; 2.02]	.69	0.61 [0.22; 1.68]	.34	1.33 [0.68; 2.60]	.40	1.11 [0.59; 2.10]	.75
3‐month cardiovascular mortality	1.54 [0.81; 2.94]	.19	1.05 [0.31; 3.59]	.94	1.73 [0.82; 3.63]	.15	1.73 [0.82; 3.63]	.15
3‐month major bleeding	0.62 [0.24; 1.47]	.30	0.68 [0.24; 1.78]	.45	0.58 [0.23; 1.48]	.26	0.64 [0.25; 1.64]	.35

Abbreviations: GPI, glycoprotein IIb‐IIIa inhibitor.

## 4 DISCUSSION

Our study showed that, in an experienced radial‐first center, the per‐procedure use of GPI, with respect to current guidelines,^1^ on top of potent recommended oral inhibitors of P2Y12, ticagrelor or prasugrel, is not associated with increased in‐hospital or 3‐month mortality or bleeding.

GPIs were used in 41% of patients, in a real‐life cohort, all pre‐treated with potent P2Y12 inhibitors. Such rates are comparable with those reported in pivotal trials PLATO‐STEMI,^10^ TRITON‐TIMI 38 STEMI^11^ and ATLANTIC trial^12^ (37%, 64%, and 43% of GPI‐use, respectively). In the more recent French FAST‐MI real‐life cohort, however, GPI were used in only 24% of patients.^13^ This “snapshot” registry, conducted over a specified 1‐month period every 5 years in France, including consecutive willing MI patients, likely leads to underrepresentation of more severe patients explaining the less use of GPI agents. Patients treated medically, or with fibrinolysis were also included, when taking into account only primary PCI patients, GPI was used in 31% of them.

### 4.1 Ischemic endpoints

The use of GPI on top of new antiplatelet regimens did not impact ischemic endpoints. The use of rapid‐onset and more potent P2Y12 inhibitors with less interpatient variability can explain the lack of benefit of GPI use that has been reported with clopidogrel in former studies.^2^, ^3^, ^4^

Similarly, in the subanalysis of the PLATO trial, the ischemic endpoints were not influenced by the administration of GPI. Interestingly, the rate of definite stent thrombosis was lower with ticagrelor versus clopidogrel in patients not receiving GPI but comparable among those receiving GPI. A possible explanation for this finding is that ticagrelor is more efficacious than clopidogrel in preventing early stent thrombosis, but the relative difference between GPI use or not becomes less relevant when ticagrelor or prasugrel are used because they already offer a rapid onset and high level of platelet aggregation inhibition. Still, in the sub‐analysis of ATLANTIC trial, stent thrombosis was numerically more frequent in the group without GPI but the difference did not reach statistical significance (1.1% vs. 0.2%, *p* = .13).^14^ Conversely our study did not show any benefit nor trend in favor of GPI to prevent stent thrombosis.

Stent thrombosis is a rare complication, especially with newer P2Y12 inhibitors. Most analyses, including ours, lack power to analyze this endpoint; however, no benefit of GPI could be found neither on other ischemic endpoints nor on mortality. One explanation to such absence of effect may be the guideline‐oriented selection of high‐risk patients who benefit from GPI use. In our study, although GRACE score was not different between the two groups, patients had more severe features in the GPI group even despite propensity matching: blood pressure was lower, cardiogenic shock requiring inotropic drugs at presentation and cardiac arrest during PCI were more frequent, crusade score was higher and initial TIMI flow grade lower. Considering such high‐risk features in those receiving GPI, it may be speculated that the absence of difference in rates of mortality could be a potential benefit of GPI use in high‐risk patients. A meta‐analysis showing the benefit of GPI use in terms of mortality associated to patient's risk profile supports such hypothesis.^4^ However, we need to be cautious in interpreting these results as our study lacks power to demonstrate a potential benefit of GPI. Moreover, despite propensity matching, our groups are not comparable, and the use of GPI was left to the discretion of the operator. As a consequence, GPI patients were probably selected and might have been at lower bleeding risk (despite similar crusade scores) and at higher thrombotic risk with a global potential benefit higher than in patients untreated by GPI. Our data support the fact that GPI may be used safely in carefully selected patients, at low bleeding and high thrombotic risks.

Current guidelines also recommend GPI treatment in P2Y12 naïve STEMI patients at the time of PCI or “early presenters.” The theoretic short delay of action of ticagrelor and prasugrel has been challenged in recent studies, especially in STEMI patients. Several studies have shown that effective platelet inhibition was achieved in only half of patients 2 h after P2Y12 administration and at least 4 h were required in a majority of patients.^15^, ^16^ In the ATLANTIC trial antiplatelet inhibition was efficient only 3 h after the loading dose in the prehospital group and after 7 h in patients who received in‐hospital loading dose.^17^ These findings support the potential benefit of GPI in the setting of P2Y12 naïve patients. The relative long FMC to balloon time in our real‐life cohort (140.59 ± 184.18 min), explained by local conditions (rural area), leaving more time to P2Y12 inhibitors to achieve adequate platelet inhibition, may also be one explanation for the absence of benefit of GPI administration.

### 4.2 Bleeding outcomes

A number of clinical trials, prior to routine pretreatment with potent P2Y12 inhibitors, have documented clinical benefits of GPI in STEMI patients, in association with clopidogrel, at the cost of a higher risk of bleeding.^2^, ^3^, ^4^ More potent P2Y12 inhibitors, have shown significant benefits in terms of survival and ischemic endpoints and are recommended over clopidogrel in STEMI patients.^18^, ^19^ However, such treatments have also been associated with an increased risk of major bleeding.^18^, ^19^ Hence the association of potent P2Y12 inhibitors and GPI may seem unnecessary or even deleterious.

To date, no clinical study has specifically assessed the benefit or risk of GPI inhibitors in combination with prasugrel or ticagrelor. Subgroup publications of the PLATO and TRITON‐TIMI 38 trials and a meta‐analysis showed that the benefit of ticagrelor and prasugrel versus clopidogrel was independent of GPI use.^20^, ^21^, ^22^ The combination of GPI with any oral antiplatelet regimen was consistently associated with a higher risk of major bleeding regardless of which P2Y12 inhibitor used. However, the magnitude of GPI‐related bleeding‐risk increase seemed to be lower with new regimens (RR 1.27 for recent ‐ticagrelor or prasugrel‐ and RR 2.01 for standard regimens ‐clopidogrel‐). Interestingly, in concordance with our findings, in the PCI subgroups, GPI use was associated with a greater risk of major bleeding, statistically significant in combination with clopidogrel but not with more recent drugs. One explanation for these findings would be that GPIs provide a high level of platelet inhibition and therefore lead to an increased rate of bleeding complications with clopidogrel. The use of more potent platelet inhibitors than clopidogrel, associated with higher rates of bleeding in patients at high risk, may reduce the differential impact of GPI use on bleeding as a consequence of concurrent risks. Such findings support the relative safety of GPI use in combination with recent regimens. In the observational TRANSLATE‐ACS study, planned GPI use, regardless of P2Y12 inhibitor type, was not associated with a significant difference in MACE but was associated with increased odds of BARC ≥2 bleeding. Similarly, the difference did not reach statistical significance in patients with prasugrel or ticagrelor.^23^ The latter study focused on planned GPI while our study included all GPI indications (i.e., routine and bailout). It is plausible, as suggested in this article and in line with our findings and current guidelines^1^ that the benefit/risk balance of GPI is in favor of a use in bail‐out situations.

Unlike other studies^14^, ^20^, ^21^, ^22^, ^23^ we did not find an increase in the risks of in‐hospital or 3‐month major (BARC ≥2) bleeding associated with GPI use. Several reasons may explain these findings. First, as GPI administration was left to the discretion of the operators, patients at lower risk of bleeding may have been preferentially selected. However, this hypothesis is not supported by the higher CRUSADE scores in the GPI group. Second, the almost exclusive use of radial access in our cohort (98% in our cohort vs. 68% in the ATLANTIC subanalysis and 88% of femoral access in the TRANSLATE ACS subanalysis)^14^, ^23^ may explain these differences, since the use of radial artery access is associated with both less bleeding complications and lower mortality in the setting of STEMI.^14^, ^24^, ^25^, ^26^ Finally, following our local STEMI protocol, enoxaparin, which has been shown to be superior to UFH in reducing ischemic endpoints, death and major bleeding, especially in STEMI patients,^27^, ^28^ was predominantly used as the adjunctive anticoagulant therapy (96% in our cohort vs. 27% in the ATLANTIC subanalysis).^14^

### 4.3 Limitations

Our data were collected as part of a single tertiary center observational quality control prospective registry. The use of a GPI was not randomized and was left to the discretion of the operators at the time of PCI. The decision to use GPI may have been influenced by multiple known or unknown variables not included in the analysis and the study may lack power to detect some significant associations. Despite multivariable adjustments and propensity score matching to blunt differences in our study groups, several variables remained significantly different between groups and unmeasured confounding factors may not be excluded. The patient population treated by newer P2Y12 inhibitors is already selected because of a lower bleeding risk as compared to those treated with clopidogrel. Hence, our results may not apply to patients at high bleeding risk. Finally, our data are limited to a single center which affects the generalizability of our findings.

## 5 CONCLUSION

Our study shows that guideline‐oriented GPI use in combination with newer fast acting potent P2Y12 inhibitors in the setting of primary PCI for STEMI in association with modern primary PCI strategies (radial access and anticoagulation with enoxaparin) may be safely used in carefully selected patients at low bleeding risk and high ischemic risk.

Further studies are needed to adequately assess the benefit of additional GPI on top of potent P2Y12 inhibitors and identify target patients.

## CONFLICT OF INTEREST

The authors have no conflicts of interest to declare that are relevant to the content of this article.

## AUTHOR CONTRIBUTIONS

All authors contributed to the study conception and design. Material preparation, data collection and analysis were performed by Thibault Heudel, Katrien Blanchart, and Farzin Beygui. The first draft of the manuscript was written by Katrien Blanchart and all authors commented on previous versions of the manuscript. All authors read and approved the final manuscript.

## Data Availability

Data available on request from the authors.

## References

[1] Ibanez B, James S, Agewall S, et al. 2017 ESC guidelines for the management of acute myocardial infarction in patients presenting with ST‐segment elevation: the task force for the management of acute myocardial infarction in patients presenting with ST‐segment elevation of the European Society of Cardiology (ESC). Eur Heart J. 2018;39:119‐177. 28886621 10.1093/eurheartj/ehx393

[2] Montalescot G, Barragan P, Wittenberg O, et al. Platelet glycoprotein IIb/IIIa inhibition with coronary stenting for acute myocardial infarction. N Engl J Med. 2001;344:1895‐1903. 11419426 10.1056/NEJM200106213442503

[3] Stone GW, Grines CL, Cox DA, et al. Comparison of angioplasty with stenting, with or without abciximab, in acute myocardial infarction. N Engl J Med. 2002;346:957‐966. 11919304 10.1056/NEJMoa013404

[4] De Luca G, Navarese E, Marino P. Risk profile and benefits from Gp IIb‐IIIa inhibitors among patients with ST‐segment elevation myocardial infarction treated with primary angioplasty: a meta‐regression analysis of randomized trials. Eur Heart J. 2009;30:2705‐2713. 19875386 10.1093/eurheartj/ehp118 PMC2777025

[5] Neumann F‐J, Sousa‐Uva M, Ahlsson A, et al. 2018 ESC/EACTS Guidelines on myocardial revascularization. Eur Heart J. 2019;40:87‐165. 30615155 10.1093/eurheartj/ehy855

[6] Mehran R, Rao SV, Bhatt DL, et al. Standardized bleeding definitions for cardiovascular clinical trials: a consensus report from the bleeding academic research consortium. Circulation. 2011;123:2736‐2747. 21670242 10.1161/CIRCULATIONAHA.110.009449

[7] Thygesen K, Alpert JS, Jaffe AS, et al. Fourth universal definition of myocardial infarction (2018). Eur Heart J. 2019;40:237‐269. 10.1093/eurheartj/ehy462 30165617

[8] Granger CB, Goldberg RJ, Dabbous O, et al. Predictors of hospital mortality in the global registry of acute coronary events. Arch Intern Med. 2003;163:2345‐2353. 14581255 10.1001/archinte.163.19.2345

[9] Subherwal S, Bach RG, Chen AY, et al. Baseline risk of major bleeding in non‐ST‐segment‐elevation myocardial infarction: the CRUSADE (can rapid risk stratification of unstable angina patients suppress ADverse outcomes with early implementation of the ACC/AHA guidelines) bleeding score. Circulation. 2009;119:1873‐1882. 19332461 10.1161/CIRCULATIONAHA.108.828541 PMC3767035

[10] Steg PG, James S, Harrington RA, et al. Ticagrelor versus clopidogrel in patients with ST‐elevation acute coronary syndromes intended for reperfusion with primary percutaneous coronary intervention: a platelet inhibition and patient outcomes (PLATO) trial subgroup analysis. Circulation. 2010;122:2131‐2141. 21060072 10.1161/CIRCULATIONAHA.109.927582

[11] Montalescot G, Wiviott SD, Braunwald E, et al. Prasugrel compared with clopidogrel in patients undergoing percutaneous coronary intervention for ST‐elevation myocardial infarction (TRITON‐TIMI 38): double‐blind, randomised controlled trial. Lancet. 2009;373:723‐731. 19249633 10.1016/S0140-6736(09)60441-4

[12] Montalescot G, van 't Hof AW, Lapostolle F, et al. Prehospital ticagrelor in ST‐Segment elevation myocardial infarction. N Engl J Med. 2014;371(11):1016‐1027. 10.1056/nejmoa1407024. 25175921

[13] Puymirat E, Simon T, Cayla G, et al. Acute myocardial infarction: changes in patient characteristics, management, and 6‐month outcomes over a period of 20 years in the FAST‐MI program (French registry of acute ST‐elevation or non‐ST‐elevation myocardial infarction) 1995 to 2015. Circulation. 2017;136:1908‐1919. 28844989 10.1161/CIRCULATIONAHA.117.030798

[14] Tavenier AH, Hermanides RS, Fabris E, et al. Efficacy and safety of glycoprotein IIb/IIIa inhibitors on top of Ticagrelor in STEMI: a subanalysis of the ATLANTIC trial. Thromb Haemost. 2020;120:65‐74. 31752042 10.1055/s-0039-1700546

[15] Valgimigli M, Tebaldi M, Campo G, et al. Prasugrel versus tirofiban bolus with or without short post‐bolus infusion with or without concomitant prasugrel administration in patients with myocardial infarction undergoing coronary stenting: the FABOLUS PRO (facilitation through Aggrastat by drOpping or shortening infusion line in patients with ST‐segment elevation myocardial infarction compared to or on top of PRasugrel given at loading dOse) trial. JACC Cardiovasc Interv. 2012;5:268‐277. 22440491 10.1016/j.jcin.2012.01.006

[16] Parodi G, Valenti R, Bellandi B, et al. Comparison of prasugrel and ticagrelor loading doses in ST‐segment elevation myocardial infarction patients: RAPID (Rapid activity of platelet inhibitor drugs) primary PCI study. J Am Coll Cardiol. 2013;61:1601‐1606. 23500251 10.1016/j.jacc.2013.01.024

[17] Silvain J, Storey RF, Cayla G, et al. P2Y12 receptor inhibition and effect of morphine in patients undergoing primary PCI for ST‐segment elevation myocardial infarction. The PRIVATE‐ATLANTIC study. Thromb Haemost. 2016;116:369‐378. 27196998 10.1160/TH15-12-0944

[18] Wiviott SD, Braunwald E, McCabe CH, et al. Prasugrel versus clopidogrel in patients with acute coronary syndromes. N Engl J Med. 2007;357:2001‐2015. 17982182 10.1056/NEJMoa0706482

[19] Wallentin L, Becker RC, Budaj A, et al. Ticagrelor versus clopidogrel in patients with acute coronary syndromes. N Engl J Med. 2009;361:1045‐1057. 19717846 10.1056/NEJMoa0904327

[20] O'Donoghue M, Antman EM, Braunwald E, et al. The efficacy and safety of prasugrel with and without a glycoprotein IIb/IIIa inhibitor in patients with acute coronary syndromes undergoing percutaneous intervention: a TRITON‐TIMI 38 (trial to assess improvement in therapeutic outcomes by optimizing platelet inhibition with Prasugrel‐thrombolysis in myocardial infarction 38) analysis. J Am Coll Cardiol. 2009;54:678‐685. 19679245 10.1016/j.jacc.2009.05.025

[21] Shimada YJ, Bansilal S, Wiviott SD, et al. Impact of glycoprotein IIb/IIIa inhibitors on the efficacy and safety of ticagrelor compared with clopidogrel in patients with acute coronary syndromes: analysis from the platelet inhibition and patient outcomes (PLATO) trial. Am Heart J. 2016;177:1‐8. 27297843 10.1016/j.ahj.2016.03.015

[22] Roule V, Agueznai M, Sabatier R, et al. Safety and efficacy of IIb/IIIa inhibitors in combination with highly active oral antiplatelet regimens in acute coronary syndromes: a meta‐analysis of pivotal trials. Platelets. 2017;28:174‐181. 27657930 10.1080/09537104.2016.1218453

[23] Ibrahim H, Kaltenbach LA, Hess CN, et al. Glycoprotein IIb/IIIa inhibitor use in patients with acute myocardial infarction undergoing PCI: insights from the TRANSLATE ACS study. Catheter Cardiovasc Interv off J Soc Card Angiogr Interv. 2019;93:E204‐E210. 10.1002/ccd.27816 30244509

[24] Jolly SS, Yusuf S, Cairns J, et al. Radial versus femoral access for coronary angiography and intervention in patients with acute coronary syndromes (RIVAL): a randomised, parallel group, multicentre trial. Lancet. 2011;377:1409‐1420. 21470671 10.1016/S0140-6736(11)60404-2

[25] Porto I, Bolognese L, Dudek D, et al. Impact of access site on bleeding and ischemic events in patients with non‐ST‐segment elevation myocardial infarction treated with Prasugrel: the ACCOAST access substudy. JACC Cardiovasc Interv. 2016;9:897‐907. 27151605 10.1016/j.jcin.2016.01.041

[26] Valgimigli M, Gagnor A, Calabró P, et al. Radial versus femoral access in patients with acute coronary syndromes undergoing invasive management: a randomised multicentre trial. Lancet. 2015;385:2465‐2476. 25791214 10.1016/S0140-6736(15)60292-6

[27] Collet J‐P, Huber K, Cohen M, et al. A direct comparison of intravenous enoxaparin with unfractionated heparin in primary percutaneous coronary intervention (from the ATOLL trial). Am J Cardiol. 2013;112:1367‐1372. 24012033 10.1016/j.amjcard.2013.07.003

[28] Silvain J, Beygui F, Barthélémy O, et al. Efficacy and safety of enoxaparin versus unfractionated heparin during percutaneous coronary intervention: systematic review and meta‐analysis. BMJ. 2012;344:e553. 22306479 10.1136/bmj.e553 PMC3271999

